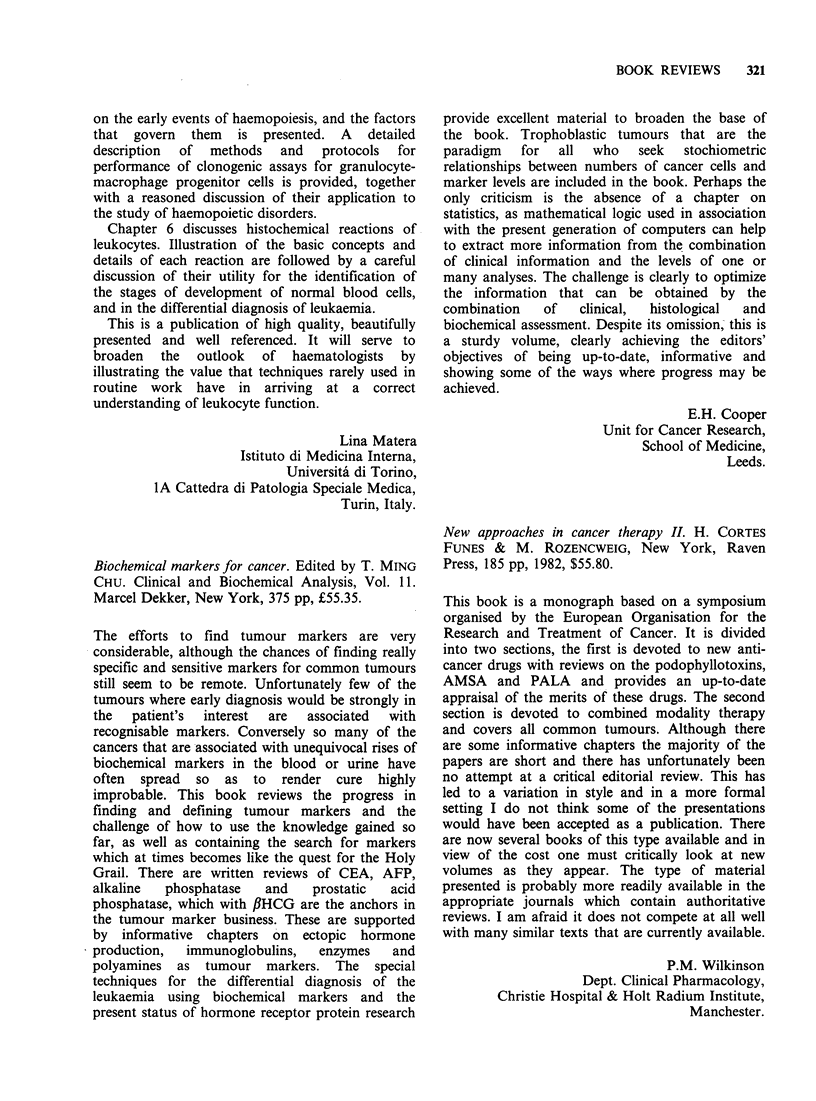# New approaches in cancer therapy II

**Published:** 1983-02

**Authors:** P.M. Wilkinson


*New approaches in cancer therapy II*. H. CORTES FUNES & M. ROZENCWEIG, New York, Raven Press, 185 pp, 1982, $55.80.

This book is a monograph based on a symposium organised by the European Organisation for the Research and Treatment of Cancer. It is divided into two sections, the first is devoted to new anti-cancer drugs with reviews on the podophyllotoxins, AMSA and PALA and provides an up-to-date appraisal of the merits of these drugs. The second section is devoted to combined modality therapy and covers all common tumours. Although there are some informative chapters the majority of the papers are short and there has unfortunately been no attempt at a critical editorial review. This has led to a variation in style and in a more formal setting I do not think some of the presentations would have been accepted as a publication. There are now several books of this type available and in view of the cost one must critically look at new volumes as they appear. The type of material presented is probably more readily available in the appropriate journals which contain authoritative reviews. I am afraid it does not compete at all well with many similar texts that are currently available.

P.M. Wilkinson
Dept. Clinical Pharmacology,
Christie Hospital & Holt Radium Institute,
Manchester.